# Synthesis, Characterization and Anti-Cancer Activity of Hydrazide Derivatives Incorporating a Quinoline Moiety

**DOI:** 10.3390/molecules21070916

**Published:** 2016-07-14

**Authors:** Murat Bingul, Owen Tan, Christopher R. Gardner, Selina K. Sutton, Greg M. Arndt, Glenn M. Marshall, Belamy B. Cheung, Naresh Kumar, David StC. Black

**Affiliations:** 1 School of Chemistry, The University of New South Wales Australia, Sydney, NSW 2052, Australia; muratbingul1983@gmail.com (M.B.); z3217087@gmail.com (C.R.G.); 2 Children’s Cancer Institute Australia for Medical Research, Lowy Cancer Research Centre, The University of New South Wales Australia, Sydney, NSW 2031, Australia; Otan@ccia.unsw.edu.au (O.T.); ssutton@ccia.unsw.edu.au (S.K.S.); garndt@ccia.unsw.edu.au (G.M.A.); gmarshall@ccia.unsw.edu.au (G.M.M.); 3 ACRF Drug Discovery Centre for Childhood Cancer, Children’s Cancer Institute Australia for Medical Research, Lowy Cancer Research Centre, The University of New South Wales Australia, Sydney, NSW 2052, Australia; 4 Kids Cancer Centre, Sydney Children’s Hospital, Randwick, NSW 2031, Australia

**Keywords:** quinoline, hydrazide-hydrazone, anticancer, neuroblastoma, breast cancer

## Abstract

Identification of the novel (*E*)-*N′*-((2-chloro-7-methoxyquinolin-3-yl)methylene)-3-(phenylthio)propanehydrazide scaffold **18** has led to the development of a new series of biologically active hydrazide compounds. The parent compound **18** and new quinoline derivatives **19**–**26** were prepared from the corresponding quinoline hydrazones and substituted carboxylic acids using EDC-mediated peptide coupling reactions. Further modification of the parent compound **18** was achieved by replacement of the quinoline moiety with other aromatic systems. All the newly synthesized compounds were evaluated for their anti-cancer activity against the SH-SY5Y and Kelly neuroblastoma cell lines, as well as the MDA-MB-231 and MCF-7 breast adenocarcinoma cell lines. Analogues **19** and **22** significantly reduced the cell viability of neuroblastoma cancer cells with micromolar potency and significant selectivity over normal cells. The quinoline hydrazide **22** also induced G_1_ cell cycle arrest, as well as upregulation of the p27^kip1^ cell cycle regulating protein.

## 1. Introduction

**Figure 1:**
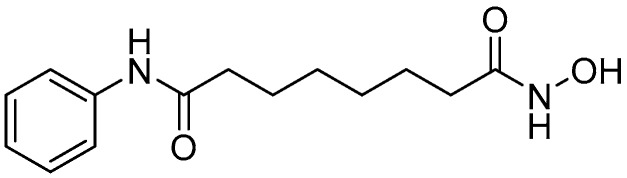
Structure of the HDACi suberoylanilide hydroxamic acid (SAHA).

Cancer is a heterogeneous type of genetic diseases, resulting from the alteration of many key genetic and molecular pathways [1]. Treatment regimens are often harsh, with conventional chemotherapies often not being effective, also inducing side-effects and encountering drug-resistance [2]. An important class of chemotherapeutics that effectively induce cell death, but can become ineffective due to drug-resistance, are the histone deacetylase inhibitors (HDACi) [3]. These molecules exert efficacy by regulating transcription of genes that suppress cell proliferation and angiogenesis, induce cell differentiation and promote apoptosis [4,5]. In particular, the HDACi suberoylanilide hydroxamic acid (SAHA, Figure 1) has shown potent cytotoxic effects towards a number of tumour types with low toxicity towards normal cells [6,7]. As SAHA targets only a few signalling pathways, single agent treatment with SAHA has been found to be ineffective against several cancers [8,9]. Cytotoxic drugs are found to be most effective when given in combination to achieve additive or synergistic effects. The combination of SAHA with other chemotherapeutic agents that possess different mechanisms of action has been considered to be a more promising approach to reduce drug resistance and minimize overlapping toxicities [8,10].

**Figure 2:**
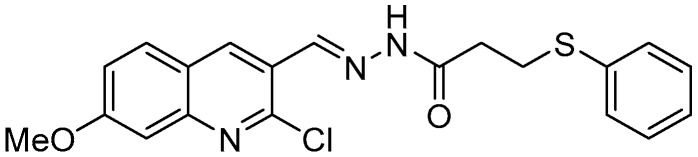
The structure of parent compound **15** from the WEHI screening.

In order to provide a basis for the discovery of novel SAHA enhancers, a subset (10,560 compounds) of the Walter & Eliza Hall Institute (WEHI) compound library was screened to identify molecules that can act synergistically with a clinical dose of SAHA to overcome resistance in SAHA-resistant MDA-MB-231 breast cancer cells, as previously reported [11]. From this screening, we identified the quinoline-hydrazide scaffold (*E*)-*N′*-((2-chloro-7-methoxyquinolin-3-yl)methylene)-3-(phenylthio)propane hydrazide (**15**, Figure 2) as a novel anti-cancer lead compound, which reduced the MDA-MB-231 cell viability by <30% in the absence of SAHA, >60% in the presence of SAHA and also displayed a differential of >55% between these conditions.

**Figure 3:**
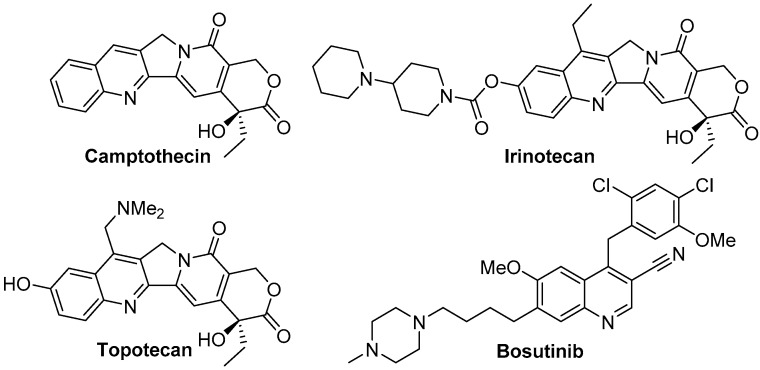
Structures of quinoline-containing anticancer drugs.

Quinoline is a pharmacologically valuable scaffold that is prevalent in a variety of biologically active synthetic and natural compounds [12]. Throughout the 20th century, the chemistry of quinolines has been the subject of intense study and different interesting bioactivity such as antibacterial, antifungal, anti-inflammatory, antimalarial and anticancer activities [13,14,15,16,17,18]. This anticancer activity is quite broad, with quinoline derivatives having been used against many cancers, such as those of the breast, prostate, gastrointestinal tract, colon and liver [19,20,21,22]. Importantly, a number of quinoline-based anticancer drugs have also been used clinically, including camptothecin and its analogues (irinotecan and topotecan) [23,24] and bosutinib (Figure 3) [25,26].

The hydrazide-hydrazone moiety (–(C=O)NHN=CH) has been identified as an important fragment for various biological activities including antibacterial, antifungal, analgesic, antiinflammatory, antidepressant and anticancer activities [27,28,29]. When used in conjunction with a quinoline system, this combination has afforded compounds with antimicrobial [30], antimycobacterial [31], anti-tubercular [32,33], anticonvulsant [34] and cytotoxic activity [35]. These biological activities are related to the many interesting and important properties of the hydrazidehydrazone moiety, such as their relatively higher metabolic stability towards proteases than amides and their tuneable, labile nature in acidic pH. The anticancer activity of quinoline-based compounds is exerted through many mechanisms, such as apoptosis [36], inhibition of angiogenesis [37], receptor inhibition and DNA intercalation [38,39,40,41]. Many compounds also exert their activity through cell cycle arrest, particularly at the G_2_/M phase [42,43,44,45] or the S phase [46,47,48]. However, a few examples have been reported where cell cycle arrest occurs at the G_0_/G_1_ phase [49,50]. p27^kip1^ is involved in the regulation of G_0_ to S phase transitions by regulating the activity of cyclin-dependent kinases [51,52]. While p27 is rarely mutated or deleted in human cancers, it is frequently deregulated through reduced expression or mislocalization to the cytoplasm [53]. Currently, p27 expression levels in cancer serve prognostic purposes [54], as they can correlate to treatment response in either a positive or inverse fashion depending upon the disease model [55,56,57,58,59,60]. However, therapeutics targeting p27 have also been investigated in numerous cancers to enforce apoptotic consequences [61,62]. In this paper, we report the quinoline-hydrazide (*E*)-*N′*-((2-chloro-7-methoxyquinolin-3-yl)methylene)-3-(phenylthio)propane hydrazide (**15**, Figure 2) as a novel anticancer lead compound. We further report the synthesis, characterization and in vitro biological evaluation of a series of quinoline-based and non-quinoline-based analogues, including their effects on cell cycle distribution and the expression of the cell cycle-related p27^kip1^ protein.

## 2. Results and Discussion

### 2.1. Synthesis of Quinoline-Based Hydrazide-Hydrazones

**Scheme 1:**
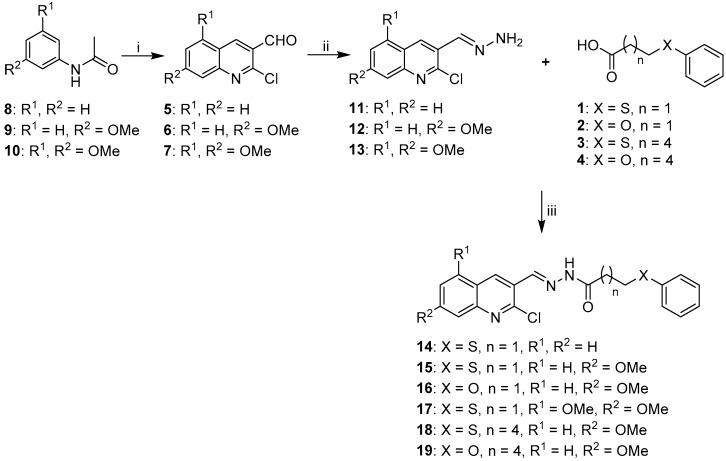
Synthesis of quinoline-based hydrazide-hydrazones. *Reagents and conditions*: i. DMF, POCl_3_, 90 °C, 12 h; ii. NH_2_NH_2_·H_2_O, EtOH, rt, 18 h. iii. EDC, DMF, Et_3_N, rt, 18 h.

In order to generate a diverse array of analogues, a convergent synthesis was envisaged, whereby the targeted hydrazide-hydrazone structure could be formed by an amide-coupling reaction between an alkyl carboxylic acid and aryl hydrazone (Scheme 1).

The alkyl acids **1**–**4** were prepared following literature methods [63]. Similarly, the substituted 2-chloro- quinoline-3-carbaldehydes **5**–**7** were isolated in yields of 47%–60% following Vilsmeier cyclisation of the corresponding acetanilides **8**–**10**, according to literature methods (Scheme 1) [64,65]. Following aqueous work-up, carbaldehydes **5**–**7** were then treated with neat hydrazine hydrate at room temperature in ethanol to generate the quinoline hydrazones **11**–**13** in 74%–87% yields (Scheme 1). Finally, the desired hydrazide-hydrazones **14**–**19** were obtained by EDC-coupling of the hydrazones (**11**–**13**) to the appropriate carboxylic acids **1**–**4** using 1-ethyl-3-(3-dimethylaminopropyl)-carbodiimide hydrochloride (EDC), which gave hydrazide-hydrazones **14**–**19** in yields of 62%–68% (Scheme 1).

### 2.2. Synthesis of Non-Quinoline Hydrazide-Hydrazones

**Scheme 2:**
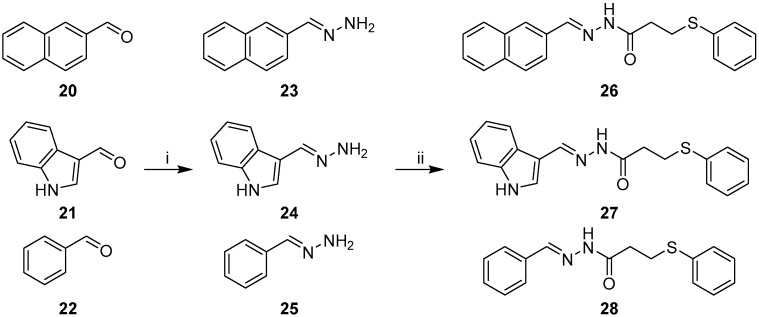
Synthesis of non-quinoline hydrazide-hydrazones. *Reagents and conditions*: i. NH_2_NH_2_·H_2_O, EtOH, rt, 18 h; ii. **11**, EDC, DMF, Et_3_N, rt, 18 h.

In order to elaborate on the structure-activity relationship and identify other heterocyclic motifs with potential cytotoxic activity, the quinoline motif was replaced with naphthalene, indole and benzaldehyde. Naphthalene was selected in order to investigate the necessity of the quinoline nitrogen, which might participate in target interaction. Indole was selected not only for its known biological activities, but also to determine if the ring size is essential for activity. Benzene was selected in order to gain insight into the necessity for a system containing a pendant ring. The aldehydes **20**–**22** were subjected to the same synthetic protocol described above, firstly being converted to the corresponding hydrazones **23**–**25**, followed by EDC-coupling to acid **11** to give the targeted hydrazide-hydrazones **26**–**28** (Scheme 2).

The new hydrazide-hydrazones **14**–**19**, **26**–**28** were characterized by physical and spectral data (IR, ^1^H-NMR, ^13^C-NMR and EIMS). The IR spectra of the derivatives showed the azomethine C–H stretching doublet absorption band at 2850–2950 cm^–1^, the C=N absorption band at 1550–1600 cm^–1^, a carbonyl stretch at 1620–1680 cm^–1^ and a strong N–H stretch at 3180–3220 cm^–1^. In the ^1^H-NMR spectra of the hydrazides, the azomethine proton appeared at 8.22–8.90 ppm as a sharp singlet, whereas the corresponding amide N–H proton appeared as a broad singlet at 9.18–9.37 ppm. The protons of the heterocyclic systems and carboxylic acid nucleus showed resonances at the expected chemical shift values.

**Figure 4:**
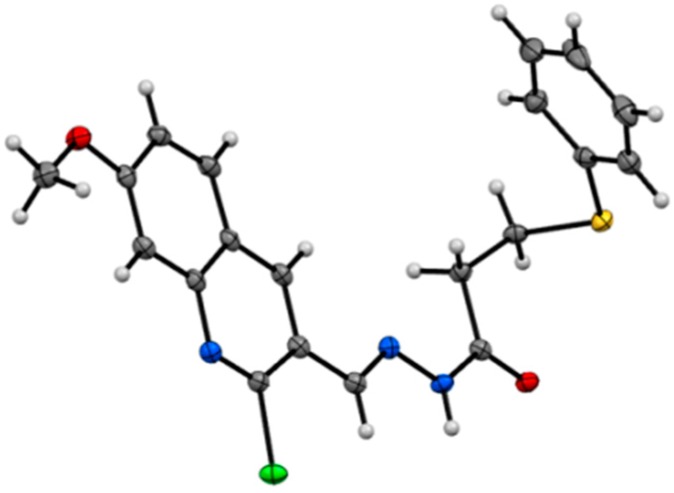
ORTEP diagram of X-ray crystal structure of **15** grown from chloroform.

As stereochemistry is an essential factor for biological activity, single crystals of **28** were examined by X-ray diffraction in order to determine the relative stereochemistry of the C=N bond (Figure 4). The X-ray diffraction study clearly indicated an *E* conformation for the C=N bond, as well as showing the alkyl chain in a bent conformation. Theoretically, this orientation might impact on the potential binding of the hydrazide-hydrazone motif to transition metals, which is a mechanism seen in multiple quinoline-hydrazone compounds [66,67,68,69]. Furthermore, it could also affect any selectivity between larger binding pockets, as opposed to shallower grooves. 

### 2.3. Biological Results

#### 2.3.1. Cell Viability Assays

**Figure 5:**
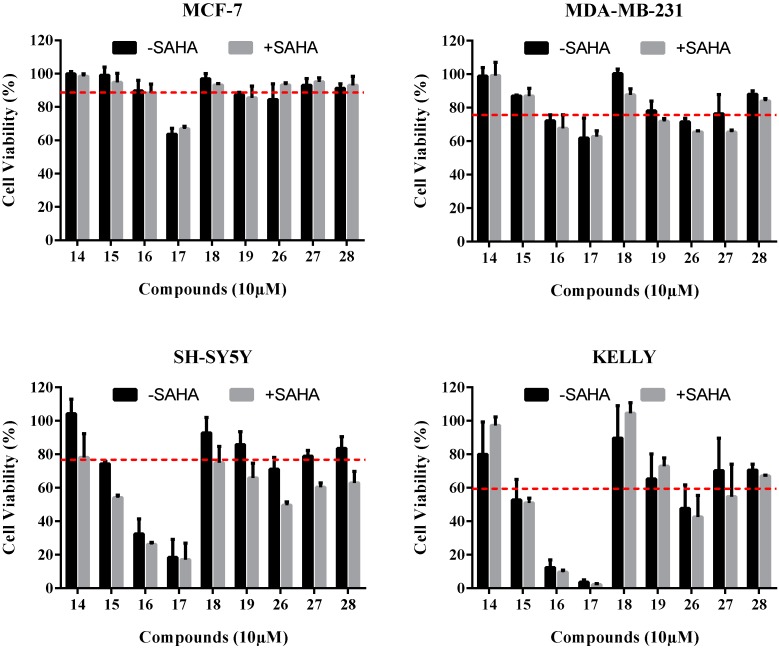
Cell viability of cancer cell lines after treatment at 10 μM for 72 h, given as a percentage of the negative control (DMSO). Red line represents the cell viability of cell lines after treatment with 1 µM SAHA (0.5 µM SAHA for KELLY cells). Data represents the mean values (±S.D.) for three independent determinations.

The newly synthesized compounds were evaluated for their ability to inhibit the growth of SH-SY5Y and Kelly neuroblastoma, and MDA-MB-231 and MCF-7 breast adenocarcinoma cell lines using the Alamar blue (Resazurin) assay [70]. Briefly, the cells were allowed to attach for 24 h in a 96-well culture plate before being exposed to the ligands at a concentration of 10 µM in DMSO for 72 h, either in the presence or absence of SAHA. Comparative values for cell viability in each well were determined by a Wallac 1420 Victor III spectrophotometer, which measured light absorbance in each well at 570 nm. The mean and standard deviation (SD) values for each compound were calculated from at least three replicate experiments. The anticancer activity of the compounds was evaluated by comparison to a negative (DMSO) control (Table 1 and Figure 5).

In general, it was found that these compounds displayed similar levels of activity whether in the presence or absence of SAHA. It was also observed that the hydrazide-hydrazone compounds had a greater cytotoxic effect on the neuroblastoma cells (SH-SY5Y and Kelly) compared to the breast cancer cell lines (MCF-7 and MDA-MB-231). MCF-7 cells were found to be the most resistant towards treatment with the hydrazide compounds, with reductions in cell viability ranging from 5%–33%. The compounds were generally more effective in the MDA-MB-231 breast adenocarcinoma cell line, with cell viability values being up to 20% lower than those in the MCF-7 cell line. Similarly, these compounds were also observed to have a greater effect on the cell viability of the Kelly neuroblastoma cell line than the SH-SY5Y cell line, again with up to 20% lower cell viability.

Notably, compound **17**, bearing an additional methoxy group, was found to be the most active agent against both breast adenocarcinoma cell lines, with cell reductions of 33% and 38% in MCF-7 and MDA-MB-231 cells, respectively. Further, this compound was also the most active agent against the neuroblastoma cell lines, with reductions in cell viability of 82% and 96% in the SH-SY5Y and Kelly cell lines, respectively. The importance of the methoxy groups was further demonstrated by the observation that compound **14**, which bears no methoxy substituents, had lower activity than the lead compound **15**. While compound **14** showed no activity towards breast adenocarcinoma cell lines, the highest activity was observed with the 20% reduction in cell viability of Kelly cells.

It was also found that replacing the sulfane linkage with an ether linkage increased the potency of the synthesized compounds. The ether **16**, which was found to be the second most active compound of those screened, displayed an enhanced potency of 42% and 40% against the SH-SY5Y and Kelly cell lines, respectively, compared to the corresponding sulfane lead compound **15**. However, only 15% and 6% of additional reductions were observed for the MDA-MB-231 and MCF-7 cell lines respectively. The ether **19** showed 7% and 25% greater reductions in cell viability towards the SH-SY5Y and Kelly cell lines, respectively, than was observed for the corresponding sulfane **18**. Similarly, 7% and 22% of additional reductions were observed for the MCF-7 and MDA-MB-231 cell lines respectively.

Hydrazides with shorter chains were also found to be more potent than their longer chain counterparts. With respect to compounds **15** (*n* = 1) and **18** (*n* = 4), bearing sulfane linkages, it was observed that compound **15** reduced viability of Kelly cells by up to 37% more than compound **18**. In the case of SH-SY5Y cell, 18% of additional reduction was observed. Similarly, the shorter chain analogue **16** reduced cell viability by up to 53% more than its longer chain analogue **19** for both Kelly and SH-SY5Y neuroblastoma cell lines. 

Additionally, the replacement of the quinoline system with other aromatic groups generally led to a reduction in activity. Replacement of the quinoline (compound **15**) with naphthalene (compound **26**) resulted in similar levels of cytotoxicity across all cell lines and exhibited up to a 54% of reduction in the case of Kelly cells. MCF-7 was found to be the most resistant cell with only 7% reduction. However, the indole (compound **27**) and phenyl (compound **28**) derivatives showed significant decrease in activity compared to **15**, with **28** typically having lower activity than **27**. This suggests that the size of this heterocycle is important for its cytotoxic activity, with bicyclic systems more favourable than the tested monocyclic counterpart.

Dose response experiments were also conducted for the most potent compounds **16** and **17**. These compounds were tested against the neuroblastoma and breast adenocarcinoma cell lines at concentrations ranging from 0.1–25.0 µM, with their IC_50_ values determined (Table 2). Compound **16** showed IC_50_ values of 5.7 and 2.4 μM against SH-SY5Y and Kelly neuroblastoma cells, respectively, with the IC_50_ value against the MCF-7 breast adenocarcinoma cell line greater than the maximum tested dosage. On the other hand, the most active compound **17**, showed IC_50_ values of 2.9 and 1.3 μM against the SH-SY5Y and Kelly cells, respectively, and values of 14.1 and 18.8 μM for MCF-7 and MDA-MB-231 breast cancer cells, respectively.

#### 2.3.2. Toxicity Study on Normal Human Cells

**Figure 6:**
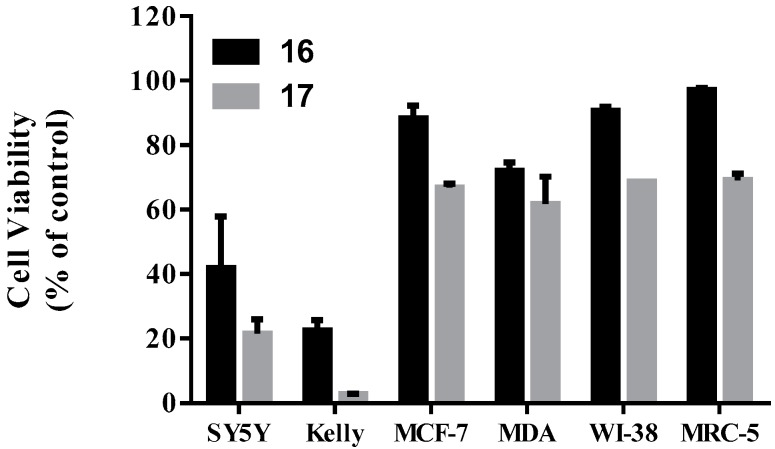
Comparative toxicity of compounds **29** and **30** against the tested cell lines at 10 µM after 72 h exposure. Cell viability values are quoted as a percentage of the negative control.

Compounds **16** and **17** were then further tested against the MRC-5 and WI-38 normal human lung fibroblast cell lines (Figure 6). At a concentration of 10 μM, **16** reduced MRC-5 and WI-38 cell viability by only 10% and 5% respectively, while **17** reduced cell viability by about 30% in the two normal lung fibroblast cell lines. Notably, these two compounds were significantly less toxic towards the normal lung fibroblast cells compared to neuroblastoma cells. Taken together, the data suggests that these novel quinoline hydrazides warrant further investigation as potential anti-cancer agents, particularly for the treatment of neuroblastoma.

#### 2.3.3. Effects on Cell Cycle and the Expression of Related Proteins

**Figure 7:**
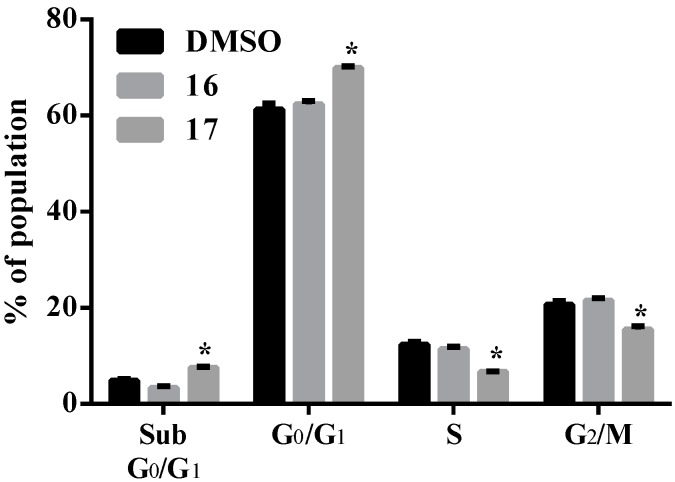
Comparative cell cycle distribution of SH-SY5Y cells after 72 h exposure to 10 µM of compounds 16 and 17. * *p* < 0.05.

In order to determine if the synthesized compounds **16** and **17** play an active role in cell cycle progression, we analysed their effect on the cell cycle progression of SH-SY5Y cells after 72 h treatment. It was found that cells treated with 10 µM of compound **16** showed no significant difference in the proportions of cells across the cell cycle, compared to the DMSO control (Figure 7). Interestingly, cells treated with 10 µM of compound **17** showed an increased proportion of cells in G_0_/G_1_, with 69% of cells in G_0_/G_1_, compared with the solvent control treated cells with 61% of cells in G_0_/G_1_ (Figure 7). Furthermore, treatment with compound **17** also reduced the number of cells in the S phase and G_2_/M phase.

**Figure 8:**
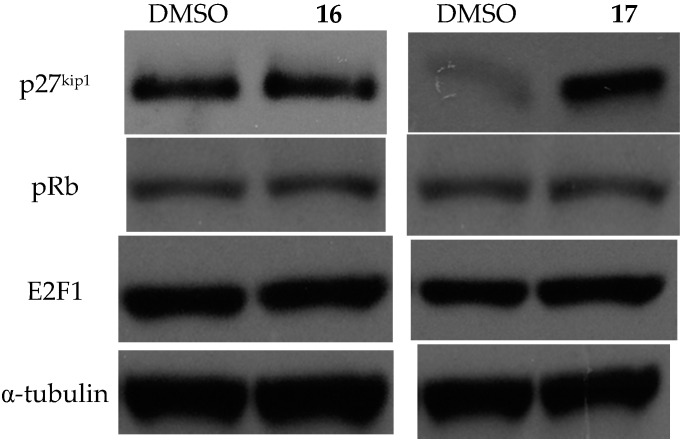
Immunoblot analysis of p27^kip1^ and E2F1 proteins isolated from SH-SY5Y cells treated with 10 µM of compounds **16** and **17** over 72 h. Anti-α-tubulin antibody was used for the loading control.

To further investigate their mechanism of action on the cell cycle, immunoblot analysis was performed on proteins harvested from SH-SY5Y cells treated with compounds **16** and **17**. No changes were observed in the levels E2F1, pRb and p27^kip1^ protein expression, in comparison to the negative control, following 72 h treatment with 10 µM of **16** (Figure 8).

On the other hand, p27^kip1^ was highly up-regulated in SH-SY5Y cells treated with 10 µM of compound **17**, in comparison with the solvent control (Figure 8). However, two other cell cycle regulatory proteins, E2F1 and pRb, were unchanged by treatment with compound **17**. Since p27^kip1^ is a protein that prevents cell progression from the G_1_ to S phase, the results suggest that compound **17** induces cell cycle arrest by up-regulating p27^kip1^.

## 3. Materials and Methods 

### 3.1. General Information

Commercially available reagents were purchased from Fluka (Sydney, NSW, Australia), Aldrich (Sydney, NSW, Australia), Acros Organics (Morris Plains, NJ, USA), Alfa Aesar (Lancashire, UK) and Lancaster (Lancashire, UK) and purified if necessary. The synthetic procedures have been reported for all compounds as general methods and appropriate references have been given for known compounds. ^1^H (300 MHz) and ^13^C-NMR (75 MHz) spectra were obtained in the designated solvents on a DPX 300 spectrometer (Bruker, Sydney, NSW, Australia). Melting points were measured using a Mel-Temp melting point apparatus and are uncorrected. Infrared spectra were recorded on a Avatar Series FTIR spectrophotometer as KBr disks (Thermo Nicolet, Waltham, MA, USA). Ultraviolet spectra were measured using a Cary 100 spectrophotometer (Varian, Santa Clara, CA, USA) in the designated solvents and data reported as wavelength (λ) in nm and adsorption coefficient (ε) in cm^−1^ M^−1^. High-resolution [ESI] mass spectra were recorded by the UNSW Bioanalytical Mass Spectrometry Facility, on an Orbitrap LTQ XL (Thermo Scientific, Waltham, MA, USA) ion trap mass spectrometer using a nanospray (nano-electrospray) ionization source.

#### 3.1.1. General Procedure for the Synthesis of Substituted Carboxylic Acids **1**–**4**

To a solution of thiophenol or phenol (10 mmol) in aqueous 10% KOH (25 mL) and EtOH (30 mL) was added 3-bromopropionic acid or 6-bromohexanoic acid (11 mmol) in a saturated solution of K_2_CO_3_ (10 mL, 5.5 mmol). The resulting mixture was heated at reflux for 5 h. The reaction mixture was neutralized with 18% HCl solution and the resulting precipitate was collected via filtration, washed with water and crystallized from petroleum ether to give the product as a white solid.

*3-(Phenylthio)propanoic acid* (**1**) [63]. White solid, yield: 88%; m.p. 59–60 °C (lit. [63] m.p. 58–59 °C); ^1^H-NMR (CDCl_3_): δ 7.41–7.33 (4H, m, ArH), 7.27–7.22 (1H, m, ArH), 3.22 (2H, t, *J* = 7.2 Hz, CH_2_), 2.65 (2H, t, *J* = 7.2 Hz, CH_2_).

*3-Phenoxypropanoic acid* (**2**) [71]. White solid, yield: 85%; m.p. 92–94 °C (lit. [71] m.p. 95–96 °C); ^1^H-NMR (CDCl_3_): δ 7.34–7.29 (4H, m, ArH), 7.01–6.92 (1H, m, ArH), 4.29 (2H, t, *J* = 6.3 Hz, CH_2_), 2.88 (2H, t, *J* = 6.3 Hz, CH_2_).

*6-(Phenylthio)hexanoic acid* (**3**) [72]. White crystals, yield: 95%; m.p. 62–63 °C (lit. [72] m.p. not given); ^1^H-NMR (CDCl_3_): δ 7.31–7.26 (2H, m, ArH), 6.96–6.90 (3H, m, ArH), 4.02 (2H, t, *J* = 7.2 Hz, CH_2_), 2.36 (2H, t, *J* = 7.5 Hz, CH_2_), 1.87–1.65 (4H, m, CH_2_-CH_2_), 1.61–1.50 (2H, m, CH_2_).

*6-Phenoxyhexanoic acid* (**4**) [72]. White crystals, yield: 89%; m.p. 66–68 °C (lit. [72] m.p. not given); ^1^H-NMR (CDCl_3_): δ 7.38–7.35 (3H, m, ArH), 7.30–717 (2H, m, ArH), 3.00 (2H, t, *J* = 6.4 Hz, CH_2_), 2.32 (2H, t, *J* = 6.2 Hz, CH_2_), 1.69–1.60 (4H, m, CH_2_-CH_2_), 1.57–1.46 (2H, m, CH_2_).

#### 3.1.2. General Procedure for the Synthesis of Acetylated Anilines **8**–**10**

A mixture of the appropriate aniline (50 mmol) and dimethoxyaminopyridine (DMAP, 50 mmol) in dichloromethane (100 mL) was stirred for 10 min at room temperature. Acetic anhydride (50 mmol) was added dropwise and the resulting mixture was stirred for a further 4 h at room temperature. Water (25 mL) was added and the resulting precipitate was thoroughly washed with ethanol followed by water to remove unreacted acetic anhydride and acetic acid. The desired product was dried at 50 °C under vacuum to yield the acetyl derivatives as a white solid.

*N-Phenylacetamide* (**8**) [73]. White solid, yield: 94%; m.p. 112–114 °C (lit. [73] m.p. 113–115 °C); ^1^H-NMR (CDCl_3_): δ 7.49 (2H, t, *J* = 4.4 Hz, H2 and H6), 7.37 (1H, bs NH), 7.34 (2H, t, *J* = 8.0 Hz, H3 and H5), 7.13 (1H, t, *J* = 7.4 Hz, H4), 2.20 (3H, s, CH_3_).

*N-(3-Methoxyphenyl)acetamide* (**9**) [74]. White solid, yield: 88%; m.p. 80–82 °C (lit. [74] m.p. 81–83 °C); ^1^H-NMR (CDCl_3_): δ 7.45 (1H, bs, NH), 7.30–7.23 (2H, m, H4–H5), 6.99 (1H, d, *J* = 8.0 Hz, H2), 6.69 (1 H, dd, *J* = 2.1, 8.1 Hz, H6), 3.82 (3H, s, OMe), 2.20 (3H, s, CH_3_).

*N-(3,5-Dimethoxyphenyl)acetamide* (**10**) [75]. White solid, yield: 90%; m.p. 152–154 °C (lit. [75] m.p. not given); ^1^H-NMR (CDCl_3_): δ 7.53 (1H, bs, NH), 6.78 (2H, d, *J* = 2.34 Hz, H2 and H4), 6.27 (1H, t, *J* = 2.34 Hz, H6), 3.80 (6H, s, 2 × OMe), 2.20 (3H, s, CH_3_)**.**

#### 3.1.3. General Procedure for the Synthesis of 2-Chloro-3-Formylquinolines (**5**–**7**).

*N*,*N*-Dimethylformamide (0.025 mol) was cooled in an ice-salt bath and treated with phosphoryl chloride (0.070 mol) for 20 min. The resulting mixture was added dropwise to a cooled solution of the appropriate acetylated aniline (**8**–**10**) (0.010 mol) in *N*,*N*-dimethylformamide (10 mL) with stirring. The mixture was stirred at 90 °C for 12 h. A small amount of crushed ice was added and the mixture was basified to pH 14 with 5 M NaOH. After stirring at ambient temperature for 1 h, the precipitate was filtered, washed with water, and dried to give the title compound.

*2-Chloroquinoline-3-carbaldehyde* (**5**) [76]. Yellow solid, yield: 60%; m.p. 150–152 °C (lit. [76] m.p. 146–149 °C); ^1^H-NMR (CDCl_3_): δ 10.59 (1H, s, CHO), 8.79 (1H, s, H4), 8.11–7.65 (4H, m, H5, H6, H7, H8).

*2-Chloro-7-methoxyquinoline-3-carbaldehyde* (**6**) [64]. Yellow solid, yield: 55%; m.p. 195–197 °C (lit. [64] m.p. 196–197 °C); ^1^H-NMR (CDCl_3_): δ 10.54 (1H, s, CHO), 8.70 (1H, s, H4), 7.89 (1H, d, *J* = 9.0 Hz, H5), 7.42 (1H, d, *J* = 2.5 Hz, H8), 7.32 (1H, dd, *J* = 2.5, 9.0 Hz, H6) 4.00 (3H, s, OMe).

*2-Chloro-5,7-dimethoxyquinoline-3-carbaldehyde* (**7**). Yellow solid, yield: 47%; m.p. 160–162 °C; UV-vis (MeOH): λ_max_ 314 nm (ε 7,100 cm^−1^ M^−1^), 347 (9,500); ^1^H-NMR (CDCl_3_): δ 11.18 (1H, s, CHO), 8.43 (1H, d, *J* = 8.7 Hz, H6), 7.32 (1H, d, *J* = 8.7 Hz, H8), 6.66 (1H, s, H4), 4.13 (6H, s, 2 × OMe); ^13^C-NMR (CDCl_3_): δ 190.18 (C=O), 164.21 (C5), 160.97 (C7), 152.89 (C2), 150.39 (aryl C), 133.91 (C4), 119.99 (C3), 113.75 (aryl C), 111.16 (C6), 92.73 (C8), 106.87 (C8), 56.66 and 56.23 (OMe) HRMS (ESI): C_12_H_10_ClNO_3_ [M]^+^ requires 274.0241, found 274.0241.

#### 3.1.4. General Procedure for the Preparation of Hydrazones **11**–**13**, **23**–**25**

A mixture of the appropriate aldehyde **5**–**7**, **20**–**22** (5 mmol) and excess hydrazine hydrate was stirred overnight in ethanol. After completion of the reaction, the solvent was evaporated. Water was added to the residue and the resulting mixture was extracted with ethyl acetate. The organic extract was dried with anhydrous sodium sulfate and the solvent was evaporated under reduced pressure to give the title compound.

*(E)-2-Chloro-3-(hydrazonomethyl)quinolone* (**11**) [77]. Yellow solid, yield: 87%; m.p. 140–142 °C (lit. [77] m.p. 135 °C); ^1^H-NMR (CDCl_3_): δ 8.63 (1H, s, H4), 8.09–8.06 (1H, m, H5), 8.06 (1H, s, CH), 7.93–7.91 (1H, m, H8), 7.79–7.73 (1H, m, H6) 7.66–7.60 (1H, m, H7), 7.58 (2H, bs, NH_2_). 

*(E)-2-Chloro-3-(hydrazonomethyl)-7-methoxyquinoline* (**12**) [77]. Yellow solid, yield: 80%; m.p. 166–168 °C (lit. [77] m.p. 165 °C); ^1^H-NMR (CDCl_3_): δ 8.57 (1H, s, H4), 8.20 (1H, s, CH), 7.74 (1H, d, *J* = 9.0 Hz, H5), 7.34 (1H, d, *J* = 2.5 Hz, H8), 7.22 (1H, dd, *J* = 2.5, 9.0 Hz, H6) 5.86 (2H, s, NH_2_), 3.97 (3H, s, OMe).

*(E)-2-Chloro-3-(hydrazonomethyl)-5,7-dimethoxyquinoline* (**13**). Yellow solid, yield: 74%; m.p. 198–200 °C; IR (KBr): ν_max_ 3403, 2942, 1610, 1499, 1466, 1349, 1263, 1220, 1175, 1132 cm^−1^; UV-vis (MeOH): λ_max_ 315 nm (ε 2,800 cm^−1^ M^−1^), 362 (4,900); ^1^H-NMR (CDCl_3_): δ 8.44 (1H, d, *J* = 8.7 Hz, H6), 8.40 (1H, s, CH), 7.38 (1H, d, *J* = 8.7 Hz, H8), 7.02 (1H, s, H4), 6.63 (2H, bs, NH_2_), 4.08 and 3.98 (each 3H, s, OMe). ^13^C-NMR (CDCl_3_): δ 159.73 (C5), 156.24 (C7), 151.29 (C2), 147.74 (aryl C), 139.93 (CH), 133.86 (C4), 119.17 (C3), 114.03 (aryl C), 102.36 (C6), 94.04 (C8), 106.87 (C8), 56.85 and 55.86 (OMe); C_12_H_12_ClN_3_NaO_2_ [M + Na]^+^ requires 288.0516, found 288.0503.

*(E)-(Naphthalen-2-ylmethylene)hydrazine* (**23**) [78]. Yellow solid, yield: 83%; m.p. 152–154 °C (lit. [78] m.p. 148–151 °C); ^1^H-NMR (CDCl_3_): δ 7.94 (1H, s, CH), 7.91–7.83 (5H, m, ArH), 7.50–7.46 (2H, m, H3 and H4), 5.63 (2H, s, NH_2_).

*(E)-3-(Hydrazonomethyl)-1H-indole* (**24**) [79]. Yellow solid, yield: 76%; m.p. 158–160 °C (lit. [79] not given); ^1^H-NMR (CDCl_3_): δ 11.69 (1H, bs, NH), 8.92 (1H, s, CH), 8.35 (1H, dd, *J* = 0.9, 1.2 Hz, H4), 7.93 (1H, s, H2), 7.49 (1H, dd *J* = 0.9,1 .2 Hz, H7), 7.27–7.17 (2H, m, H5 and H6) 6.62 (2H, bs, NH_2_).

*(E)-Benzylidenehydrazine* (**25**) [80]. Yellow crystal, yield: 88%; m.p. 92–94 °C (lit. [80] m.p. 89–90 °C); ^1^H-NMR (CDCl_3_): δ 8.72 (1H, s, CH), 7.91–7.88 (2H, m ArH), 7.51–7.49 (3H, m, ArH), 5.63 (2H, bs, NH_2_).

#### 3.1.5. General Procedure for EDC Coupling (compounds **14**–**19**, **26**–**28**)

The appropriate hydrazone **11**–**13**, **23**–**25** (4 mmol) and substituted carboxylic acid **1**–**4** (4 mmol) and EDC (4.4 mmol) were dissolved in dry *N-N*-dimethylformamide. Triethylamine (4.4 mmol) was added and the solution was stirred overnight at room temperature. The reaction mixture was diluted with ethyl acetate (300 mL) and washed with 0.5 M hydrochloric acid solution (100 mL) and saturated NaHCO_3_ solution (100 mL). The organic phase was dried over MgSO_4_, filtered and concentrated under reduced pressure to give the title compound. 

*(E)-N′-((2-Chloroquinolin-3-yl)methylene)-3-(phenylthio)propanehydrazide* (**14**). Yellow solid, yield: 65%; log P: 4.79; m.p. 172–174 °C; IR (KBr): ν_max_ 3181, 3070, 2960, 1668, 1616, 1598, 1571, 1486,1436, 1409, 1397, 1328, 1275, 1212 cm^−1^; UV-vis (MeOH): λ_max_ 323 nm (ε 17,300 cm^−1^ M^−1^), 352 (7,900); ^1^H-NMR (CDCl_3_): δ 9.28 (1H, bs, NH), 8.61 (1H, s, H4), 8.27 (1H, s, CH), 8.06 (1H, d, *J* = 8.4 Hz, H5), 7.92 (1H, d, *J* = 8.4 Hz H8), 7.84–7.79 (1H, m, H6) 7.68–7.62 (1H, m, H7), 7.46–7.31 (5H, m, ArH ) 3.38 (2H, t, *J* = 7.3 Hz, CH_2_), 3.20 (2H, t, *J* = 7.3 Hz,, CH_2_); ^13^C-NMR (CDCl_3_): δ 173.43 (C=O), 148.94 (C2), 148.54 (aryl C), 138.96 (CH), 135.67 (C4), 131.64 (aryl C), 129.60 and 129.04 (aryl CH), 128. 50 (C7), 128.37 (aryl C), 127.77 (C5 and C8), 127.00 (C6), 126.33 (C3), 125.72 (2 × aryl CH), 32.70 (CH_2_) and 28.61 (CH_2_) HRMS (ESI): C_16_H_17_N_2_O_3_S [M]^+^ requires 285.1062, found 285.1053.

*(E)-N′-((2-Chloro-7-methoxyquinolin-3-yl)methylene)-3-(phenylthio)propanehydrazide* (**15**). Yellow solid, yield: 65%; log P: 4.66; m.p. 180–182 °C; IR (KBr): ν_max_ 3171, 3071, 2961, 1675, 1616, 1599, 1488, 1446, 1407, 1365, 1331, 1274, 1232, 1163, 1126 cm^−1^; UV-vis (MeOH): λ_max_ 324 nm (ε 15,800 cm^−1^ M^−1^), 352 (14,600); ^1^H-NMR (CDCl_3_): δ 9.37 (1H, bs, NH), 8.51 (1H, s, H4), 8.24 (1H, s, CH), 7.79 (1H, d, *J* = 9.0 Hz, H5), 7.44 (1H, d, *J* = 2.4 Hz, H8), 7.32–7.26 (5H, m, ArH), 7.23 (1H, dd, *J* = 2.4, 9.0 Hz, H6), 3.99 (3H, s, OMe), 3.37 (1H, t, *J* = 7.2 Hz, CH_2_), 3.19 (1H, t, *J* = 7.2 Hz, CH_2_); ^13^C-NMR (CDCl_3_): δ 171.21 (C=O), 162.57 (C2), 150.02 (aryl C), 149.46 (C7), 139.45 (CH), 135.78 (C4), 135.22 (aryl C), 129.54 (C5), 129.47, 129.41, 129.20, 129.02, and 126.28 (aryl CH), 123.02 (aryl C), 122.26 (C3), 121.14 (C6), 106.61 (C8), 55.76 (OMe), 32.69 (CH_2_) and 28.62 (CH_2_) HRMS (ESI): C_21_H_20_ClN_2_O_2_SNa [M + Na]^+^ requires 422.0706, found 422.0697.

*(E)-N′-((2-Chloro-7-methoxyquinolin-3-yl)methylene)-3-phenoxypropanehydrazide* (**16**). Yellow solid, yield: 67%; log P: 4.1; m.p. 206–208 °C; IR (KBr): ν_max_ 3186, 3065, 2932, 1674, 1619, 1600, 1489, 1458, 1400, 1365, 1334, 1292, 1237, 1163, 1127 cm^−1^; UV-vis (MeOH): λ_max_ 322 nm (ε 17,500 cm^−1^ M^−1^), 350 (16,800); ^1^H-NMR (CDCl_3_): δ 9.30 (1H, bs, NH), 8.63 (1H, s, H4), 8.26 (1H, s, CH), 7.81 (1H, d, *J* = 9.0 Hz, H5), 7.40–7.20 (5H, m, ArH), 7.00 (1H, d, *J* = 2.4 Hz, H8), 6.98 (1H, dd, *J* = 2.4, 9.0 Hz, H6), 4.47 (1H, t, *J* = 7.2 Hz, CH_2_), 3,99 (3H, s, OMe), 3.37 (1H, t, *J* = 7.2 Hz, CH_2_); ^13^C-NMR (CDCl_3_): δ 172.65 (C=O), 162.59 (C2), 158.59 (aryl C), 150.04 (aryl C), 149.47 (C7), 139.40 (CH), 135.26 (C4), 129.75 (C5), 129.52, 129.44 (aryl CH) and 123.02 (aryl C), 122.31 (C3), 121.15 (aryl CH), 121.00 (C6), 114.64 (aryl CH), 106.61 (C8), 63.22 (CH2), 55.76 (OMe), 32.87 (CH_2_) HRMS (ESI): C_21_H_21_ClN_2_O_3_ [M + H]^+^ requires 384.1115, found 384.1105.

*(E)-N′-((2-chloro-5,7-dimethoxyquinolin-3-yl)methylene)-3-(phenylthio)propanehydrazide* (**17**). Yellow solid, yield: 63%; log P: 4.54; m.p. 168–170 °C; IR (KBr): ν_max_ 3174, 3074, 2940, 1666, 1612, 1585, 1566, 1497, 1479, 1448, 1398, 1367, 1346, 1275, 1239 cm^−1^; UV-vis (MeOH): λ_max_ 313 nm (ε 8,700 cm^−1^ M^−1^), 356 (8,000); ^1^H-NMR (CDCl_3_): δ 8.80 (1H, s, CH), 8.58 (1H, bs, NH) 8.44 (1H, d, *J* = 8.1 Hz, H6), 8.41 (1H, d, *J* = 8.1 Hz, H8), 7.45–7.42 (2H, m, ArH), 7.32–7.26 (3H, m, ArH), 6.70 (1H, s, H4), 4.09 and 4.00 (each 3H, s, OMe), 3.41 (2H, t, *J* = 7.2 Hz, CH_2_), 3.17 (2H, t, *J* = 7.2 Hz,, CH_2_); ^13^C-NMR (CDCl_3_): δ 172.89 (C=O), 160.85 (C7), 157.56 (C5), 152.15 (C2), 148.65 (aryl C), 139.93 (CH), 135.92 (aryl C) 134.01 (C4), 129.47, 129.21, 128.28 (aryl CH), 125.59 (aryl CH), 119.56 (C3), 114.36 (aryl C), 108.54 (C6), 93.74 (C8), 56.65 (OMe), 56.01 (OMe), 33.65 (CH_2_) and 28.44(CH_2_)HRMS (ESI): C_21_H_20_ClN_3_O_3_SNa [M + Na]^+^ requires 452.0812, found 452.0806.

*(E)-N′-((2-Chloro-7-methoxyquinolin-3-yl)methylene)-6-(phenylthio)hexanehydrazide* (**18**). Yellow solid, yield: 68%; log P: 5.78; m.p. 138–140 °C; IR (KBr): ν_max_ 3202, 3052, 2928, 1669, 1622, 1597, 1566, 1550, 1491, 1479, 1465, 1436, 1363, 1334, 1260 cm^−1^; UV-vis (MeOH): λ_max_ 322 nm (ε 18,100 cm^−1^ M^−1^), 352 (16,900); ^1^H-NMR (CDCl_3_): δ 9.18 (1H, bs, NH), 8.63 (1H, s, H4), 8.22 (1H, s, CH), 7.81 (1H, d, *J* = 9.0 Hz, H5), 7.40–7.20 (5H, m, ArH), 7.19 (1H, d, *J* = 2.4 Hz, H8), 7.18 (1H, dd, *J* = 2.4, 9.0 Hz, H6), 3,99 (3H, s, OMe), 2.99 (2H, t, *J* = 7.6 Hz, CH_2_), 2.84 (2H, t, *J* = 7.6 Hz, CH_2_), 1.85–1.73 (4H, m, CH_2_-CH_2_), 1.67–1.60 (4H, m, CH_2_); ^13^C-NMR (CDCl_3_): δ 175.42 (C=O), 162.52 (C2), 149.96 (aryl C), 149.46 (C7), 138.79 (CH), 136.76 (C4), 135.10 (aryl C), 129.42 (C5), 129.06, 128.94, 128.86, 125.77 (aryl CH), 123.23 (aryl C), 122.33 (C3), 121.10 (C6), 106.60 (C8), 55.76 (OMe), 33.46 (CH2), 32.50 (CH_2_), 28.90 (CH_2_) 28.48, 28.28 and 24.08(CH_2_) HRMS (ESI): C_23_H_24_ClN_3_O_2_SNa [M + Na]^+^ requires 464.1163, found 464.1170.

*(E)-N′-((2-Chloro-7-methoxyquinolin-3-yl)methylene)-6-phenoxyhexanehydrazide* (**19**). Yellow solid, yield: 62%; log P: 5.21; m.p. 134–136 °C; IR (KBr): ν_max_ 3191, 3082, 2932, 1667, 1618, 1490, 1467, 1399, 1337, 1286, 1232, 1164, 1127, 1105, 1081 cm^−1^; UV-vis (MeOH): λ_max_ 323 nm (ε 18,200 cm^−1^ M^−1^), 350 (17,200); ^1^H-NMR (CDCl_3_): δ 9.32 (1H, bs, NH), 8.63 (1H, s, H4), 8.24 (1H, s, CH), 7.79 (1H, d, *J* = 9.0 Hz, H5), 7.35 (1H, d, *J* = 2.4 Hz, H8), 7.31–7.23 (5H, m, ArH), 6.92 (1H, dd, *J* = 2.4, 9.0 Hz, H6), 4.02 (2H, t, *J* = 7.6 Hz, CH_2_), 3,99 (3H, s, OMe), 2.88 (2H, t, *J* = 7.6 Hz, CH_2_), 1.94–1.87 (4H, m, CH_2_-CH_2_), 1.67–1.65 (4H, m, CH_2_); ^13^C-NMR (CDCl_3_): δ 175.54 (C=O), 162.50 (C2), 159.03 (aryl C), 149.95 (aryl C), 149.46 (C7), 138.79 (CH), 135.09 (C4), 129.43 ( C5) and 123.26 (aryl C), 122.33 (C3), 121.09 (2 × aryl CH), 120.56 (C6), 114.47 (2 × aryl CH), 106.59 (C8), 67.61 (CH2), 55.75 (OMe), 32.87 (CH_2_), 29.15, 25.91 and 24.33 (CH_2_) HRMS (ESI): C_23_H_25_ClN_3_O_3_ [M + H]^+^ requires 426.1575, found 426.1579.

*(E)-N'-(Naphthalen-2-ylmethylene)-3-(phenylthio)propanehydrazide* (**26**). Yellow solid, yield: 72%; log P: 4.8; m.p. 166–168 °C; IR (KBr): ν_max_ 3325, 3276, 1686, 1550, 1501, 1441, 1284, 1220, 1237, 1002, 832, 719, 760 cm^−1^; UV-vis (MeOH): λ_max_ 300 nm (ε 30,300 cm^−1^ M^−1^), 309 (36,100); ^1^H-NMR (CDCl_3_): δ 9.40 (1H, s, CH), 7.94–7.87 (5H, m, ArH,), 7.58–7.55 (2H, m, H6 and H7 npthl.), 7.49–7.46 (2H, m, ArH), 7.37–7.32 (2H, m, ArH). 7.28–7.25 (1H, m, ArH), 3.38 (2H, t, *J* = 7.2 Hz, CH_2_), 3.21 (2H, t, *J* = 7.2 Hz, CH_2_); ^13^C-NMR (CDCl_3_): δ 173.73 (C=O), 143.98 (CH), 135.84 (aryl C), 134.32 (aryl C), 133.12 (aryl C), 129.79 and 129.04 (2 × aryl CH), 128.70 (C2), 128.37 (C5), 127.93 (C8), 127.21 (C4), 126.75 (C1 and C3), 126.33 (C6 and C7), 122.71 (aryl CH), 32.97 (CH_2_) and 28.90 (CH_2_) HRMS (ESI): C_20_H_19_N_2_OS [M + H]^+^ requires 335.1218, found 335.1213.

*(E)-N'-((1H-Indol-3-yl)methylene)-3-(phenylthio)propanehydrazide* (**27**). Yellow solid, yield: 58%; log P: 3.35; m.p. 226–228 °C; IR (KBr): ν_max_ 3385, 3134, 3060, 2917, 1721, 1624, 1521, 1490, 1448,1438, 1390, 1364, 1330, 1299, 1231 cm^−1^; UV-vis (MeOH): λ_max_ 344 nm (ε 38,600 cm^−1^ M^−1^), ^1^H-NMR (CDCl_3_): δ 9.04 (1H, bs, NH), 8.91 (1H, bs, NH), 8.88 (1H, s, CH), 8.48 (1H, s, H2), 8.45 (1H, dd, *J* = 0.9, 1.2 Hz, H4), 8.29 (1H, dd *J* = 0.9, 1.2 Hz, H7), 7.51–7.41 (5H, m, ArH) 7.36–7.23 (2H, m, H5 and H6), 3.48 (2H, t, *J* = 7.2 Hz, CH_2_), 3.32 (2H, t, *J* = 7.2 Hz, CH_2_); ^13^C-NMR (CDCl_3_): δ 170.93 (C=O), 153.95 (CH), 137.74 (aryl C), 136.52 (aryl C), 131.06 (C2), 129.67 (2 × aryl CH), 128.59 (2 × aryl CH), 125.45 (aryl C), 126.34 (CH), 121.78 (C4 and C6), 117.98 (C5), 112.57 (C7), 112.20 (C3) 36.80 (CH_2_) and 27.51 (CH_2_) HRMS (ESI): C_18_H_19_N_3_OS [M + H]^+^ requires 324.1171, found 324.1163.

*(E)-N'-Benzylidene-3-(phenylthio)propanehydrazide* (**28**). Yellow solid, yield: 68%; log P: 3.81; m.p. 104–106 °C; IR (KBr): ν_max_ 3176, 3089, 2965, 1667, 1607, 1583, 1571, 1481, 1438, 1423, 1356, 1315, 1298, 1263 cm^−1^; UV-vis (MeOH): λ_max_ 301 nm (ε 11,000 cm^−1^ M^−1^); ^1^H-NMR (CDCl_3_): δ 10.21 (1H, bs, NH), 7.87 (1H, s, CH), 7.65–7.62 (2H, m ArH), 7.47 (1H, m, ArH), 7.45–7.42 (4H, m, ArH), 7.36–7.24 (3H, m, ArH), 3.35 (2H, t, *J* = 7.2 Hz, CH_2_), 3.16 (2H, t, *J* = 7.2 Hz, CH_2_); ^13^C-NMR (CDCl_3_): δ 174.37 (C=O), 144.40 (CH), 135.85 (aryl C), 133.70 (C1), 130.23 (C5), 129.78 (C2 and C3), 129.02 (aryl C), 128.75 (C4 and C6), 127.26 (2 × aryl CH), 126.31 (2 × aryl CH), 32.97 and 28.97 (CH_2_) HRMS (ESI): C_16_H_16_N_2_OSNa [M + Na]^+^ requires 307.0881, found 307.0872.

### 3.2. Cell Biology Techniques

The SH-SY5Y and Kelly human neuroblastoma cell lines were generously donated by Dr. J. Biedler (Memorial Sloan-Kettering Cancer Center, New York, NY, USA). The MDA-MB-231 and MCF-7 breast cancer cell lines were purchased from the American Type Culture Collection. All cell lines were cultured under standard conditions at 37 °C in 5% CO_2_ as an adherent monolayer in Dulbecco’s modified Eagle’s medium supplemented with l-glutamine (DMEM) (Invitrogen, Waltham, MA, USA) and 10% fetal calf serum (FCS) (Thermo Fisher Scientific, Waltham, MA, USA).

#### 3.2.1. Method for Cell Viability Assays

Cell viability was measured by the standard Alamar blue assay, as previously described [34]. Briefly, cells were allowed to attach for 24 h in 96-well culture plates. The cells were then continuously exposed to serial dilutions of the hydrazide-hydrazone derivatives for 72 h, either in the presence or absence of SAHA (0.5 or 1 µM), with five replicate wells for each determination. Cell viability was determined by the addition of 22 µL of Alamar blue reagent, recorded at comparative 0 h and 5 h values, using a Wallac 1420 Victor III spectrophotometer (GMI, Ramsey, MN, USA), which measured light absorbance in each well at 570 nm. The cell viability of each plate was calculated as a percentage compared to matched DMSO controls (0.5%). The mean (+/−SEM) is shown for three independent experiments.

#### 3.2.2. Cell Cycle Study by Propidium Iodide Staining

The cell cycle phase distribution of a cell population was determined by measuring cell DNA content using flow cytometry with propidium iodide (PI) (Sigma Aldrich) staining. The cells were trypsinized and resuspended at a concentration of 1 × 10^6^ in a complete medium, then pelleted by cold centrifugation at 1200 r.p.m., rinsed with PBS and recollected as a cell pellet. The cells were then resuspended in 0.1 mL cold PBS and were fixed in 80% cold ethanol by adding 1 mL on top of the cell suspension and incubated at 4 °C for 30 min. Prior to flow cytometric analysis, the cells were pelleted and the entire supernatant carefully discarded. The samples were treated with RNase A (Sigma, Sydney, NSW, Australia) for 30 min at room temperature and then stained with propidium iodide (50 μg/mL, final concentration) for 45 min at room temperature. The samples were analysed using a BD Caliber Flow cytometer (BD, San Jose, CA, USA). The cell cycle analysis was performed in FlowJo. Cell populations were divided into three subsets of cells that represented the sub G_0_/G_1_, G_0_/G_1_ phase, S phase and G_2_/M (as determined from comparison with untreated cell control gating).

#### 3.2.3. Immunoblotting of Cell Cycle Regulatory Protein Expression

Whole-cell lysates were prepared in RIPA buffer as described [81]. 20 µg of lysates were loaded onto Criterion Gels TRIS-HCl 10%–14% Polyacrylamide gels (Bio-Rad, NSW, Australia). Antibodies used were p27^kip1^ (1/500, BD), pRb (1/500, Cell Signaling, San Jose, CA, USA), E2F1 (1/500, Zymed, Waltham, MA, USA), and α-tubulin (1/2000, Sigma-Aldrich, Sydney, NSW, Australia) as loading control.

### 3.3. Statistical Analysis

Results of the cell viability studies were statistically analyzed using the two-tailed, unpaired Student’s t-test. Results are expressed as mean values with 95% confidence intervals.

## 4. Conclusions

The quinoline-hydrazide-hydrazone motif was identified in a library screen as a promising lead scaffold for anti-cancer activity against neuroblastoma cells. This work describes the synthesis of a series of related quinoline hydrazide-hydrazones as well as three derivatives containing different aromatic systems, via an EDC-mediated coupling. The methodology involved the simple reaction of hydrazones with substituted carboxylic acids under EDC-mediated conditions. Preliminary SAR investigations revealed that the presence of a bicyclic system comprised of 6-membered rings is favoured for activity. Furthermore, the presence of methoxy groups was found to increase activity, as was exchange of the use of an ether linkage, rather than a sulfane. The two most potent analogues **16** and **17** showed low micromolar potency against neuroblastoma cells. Furthermore, these two compounds displayed significant selectivity for neuroblastoma cancer cells over normal human lung fibroblast cells. It was also found that compound **17** induced upregulation of the cell cycle mediator p27^kip1^, with a corresponding cell cycle arrest at G_1_ in SH-SY5Y neuroblastoma cells.

## Figures and Tables

**Table 1:** Cell viability of cancer cell lines after treatment at 10 μM for 72 h, given as a percentage of the negative control (DMSO). Data represents the mean values (±S.D.) for three independent determinations.

Compound	Cell Viability (% of Negative Control)			
	MCF-7	MDA-MB-231	SH-SY5Y	Kelly
**14**	98.5 ± 1.0	93.8 ± 3.6	104.2 ± 6.2	80.0 ± 13.7
**15**	94.8 ± 3.8	86.9 ± 0.5	74.3 ± 1.0	52.7 ± 8.7
**16**	88.4 ± 3.8	72.0 ± 2.6	32.4 ± 6.4	12.4 ± 3.2
**17**	66.8 ± 1.2	61.8 ± 8.4	18.4 ± 7.0	3.7 ± 1.0
**18**	93.1 ± 0.6	100.2 ± 2.0	92.8 ± 6.6	89.7 ± 13.7
**19**	85.6 ± 4.9	78.0 ± 4.1	85.8 ± 5.5	65.2 ± 10.5
**26**	93.1 ± 1.0	71.5 ± 1.5	71.0 ± 5.0	47.6 ± 9.9
**27**	95.1 ± 1.7	76.4 ± 8.0	78.8 ± 2.5	70.2 ± 13.7
**28**	93.1 ± 3.8	87.9± 1.5	83.6 ± 4.9	70.6 ± 2.4

**Table 2:** IC_50_ values of compounds **16** and **17** against four different cancer cell lines.

Compound	IC_50_ (µM) ^a^			
	MCF-7	MDA-MB-231	SH-SY5Y	Kelly
**16**	>25.0	22.1	5.7	2.4
**17**	14.1	18.8	2.9	1.3

^a^ Compound concentration required to reduce cell viability by 50%, as determined by the Alamar Blue assay, under conditions allowing untreated controls to undergo at least five consecutive rounds of multiplication.

## Abbreviations

The following abbreviations are used in this manuscript:

EDC: 1-ethyl-3-(3-dimethylaminopropyl)carbodiimide hydrochloride
HDACi: Histone deacetylase inhibitors
SAHA: Suberoylanilide hydroxamic acid
WEHI: Walter & Eliza Hall Institute
DMF: Dimethylformamide
SD: Standart Deviation
DMSO: Dimethylsulfoxide
DMAP: Dimethoxyaminopyridine
KBr: Potassium Bromide
Et_3_N: Triethylamine
PI: Propidium iodide
SEM: Standart Error of Mean
ORTEP: Oak Ridge thermal ellipsoid
